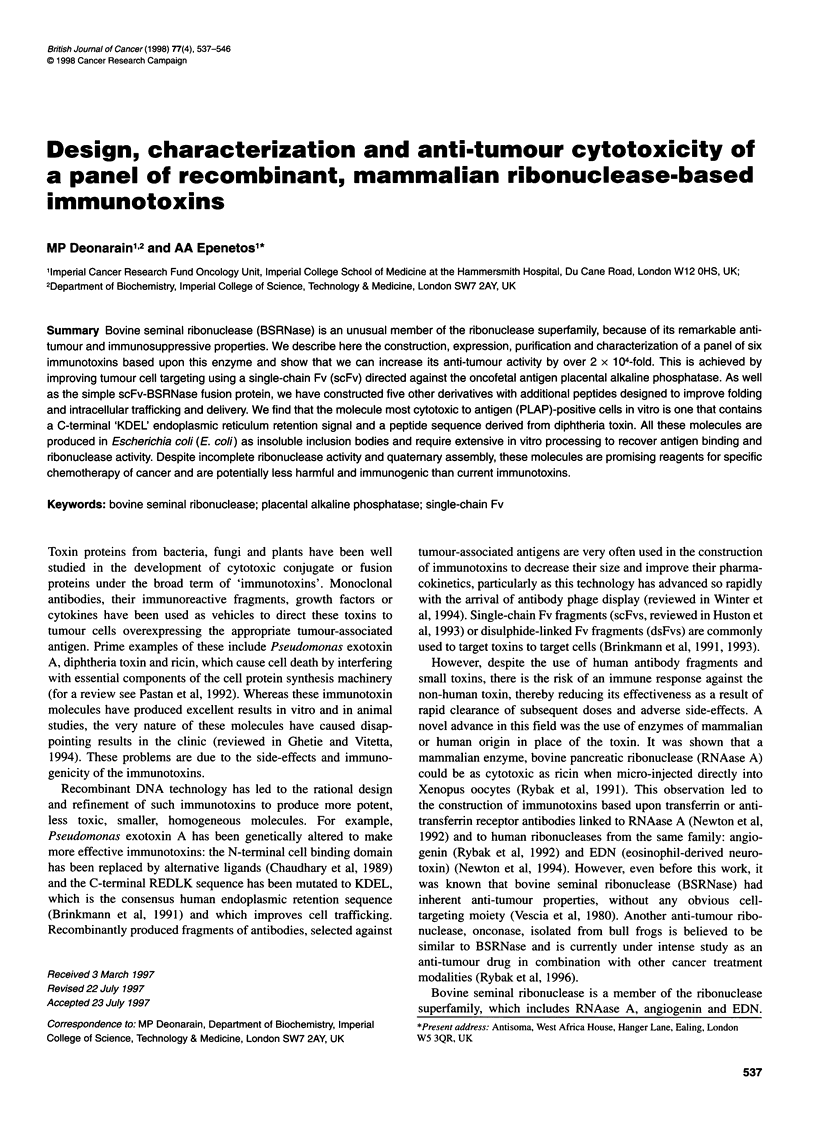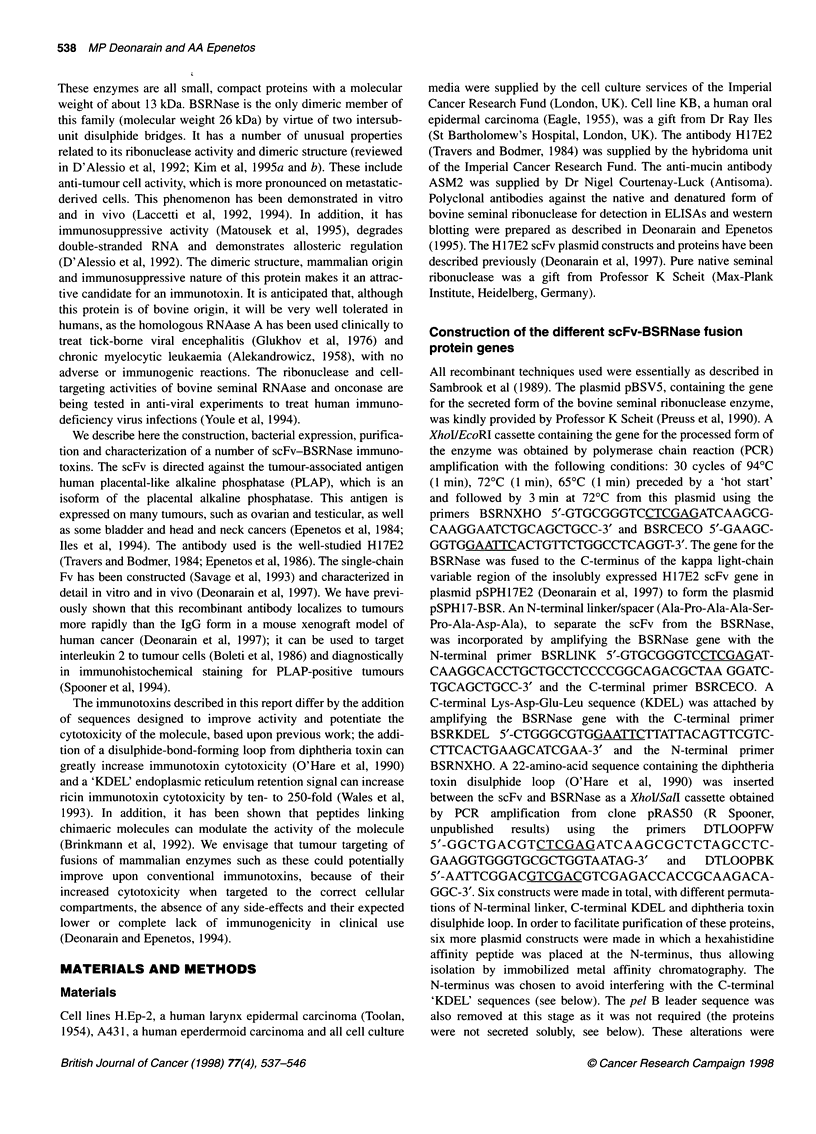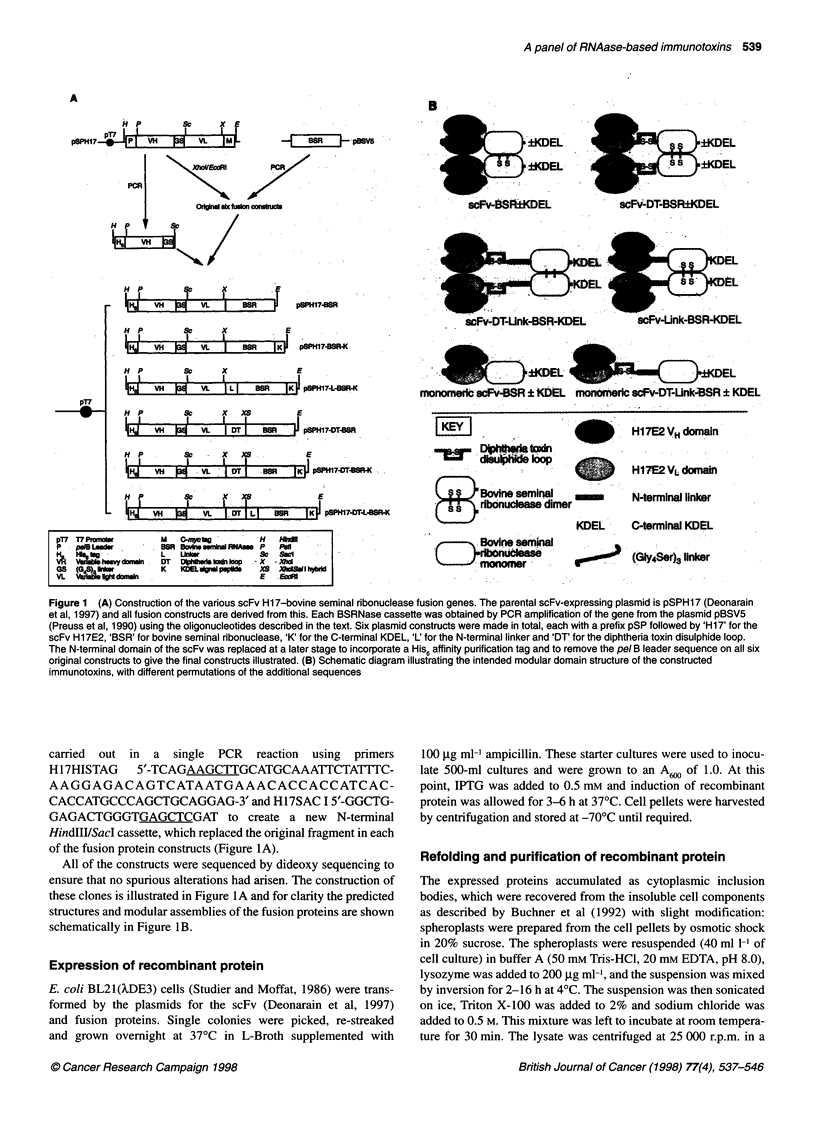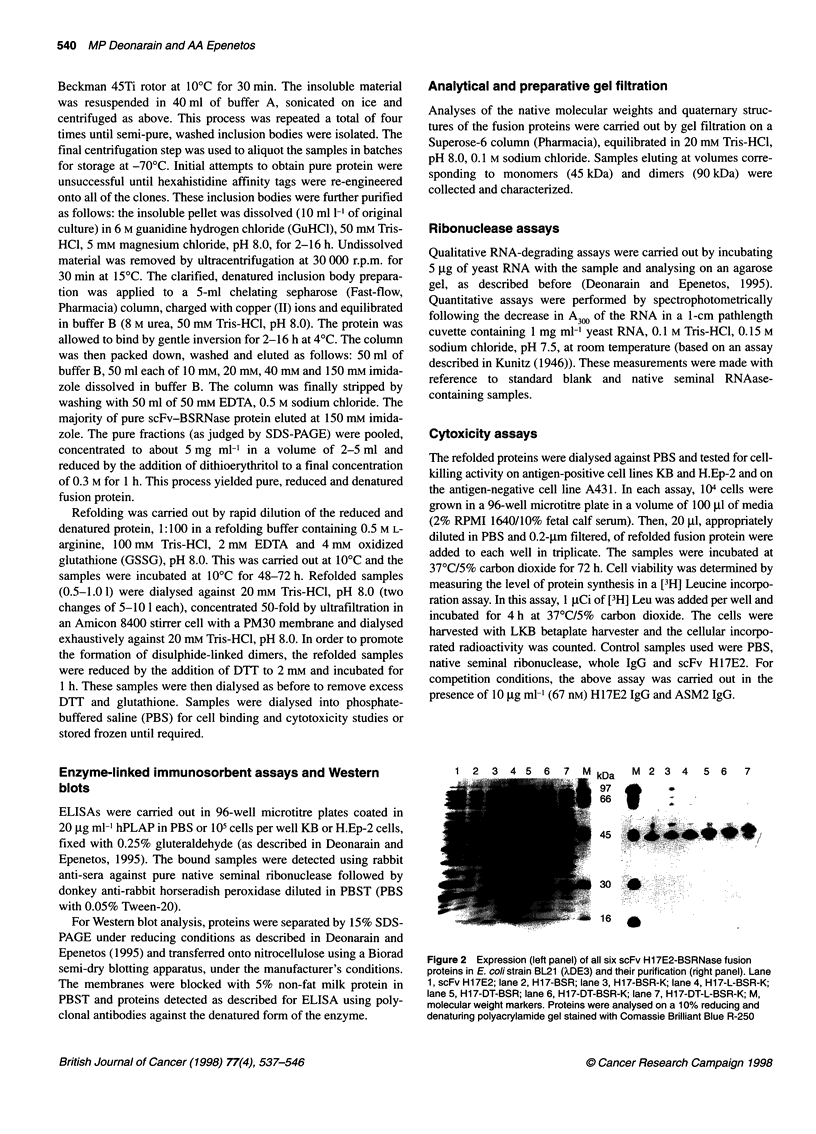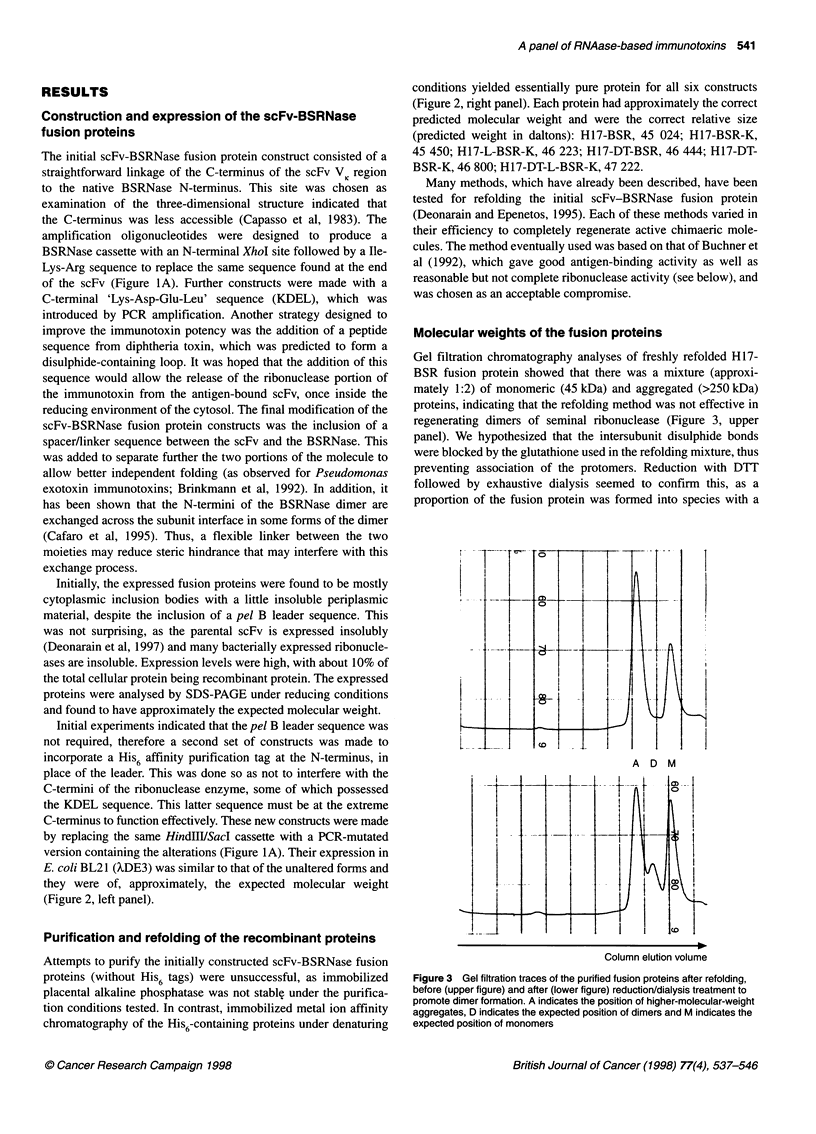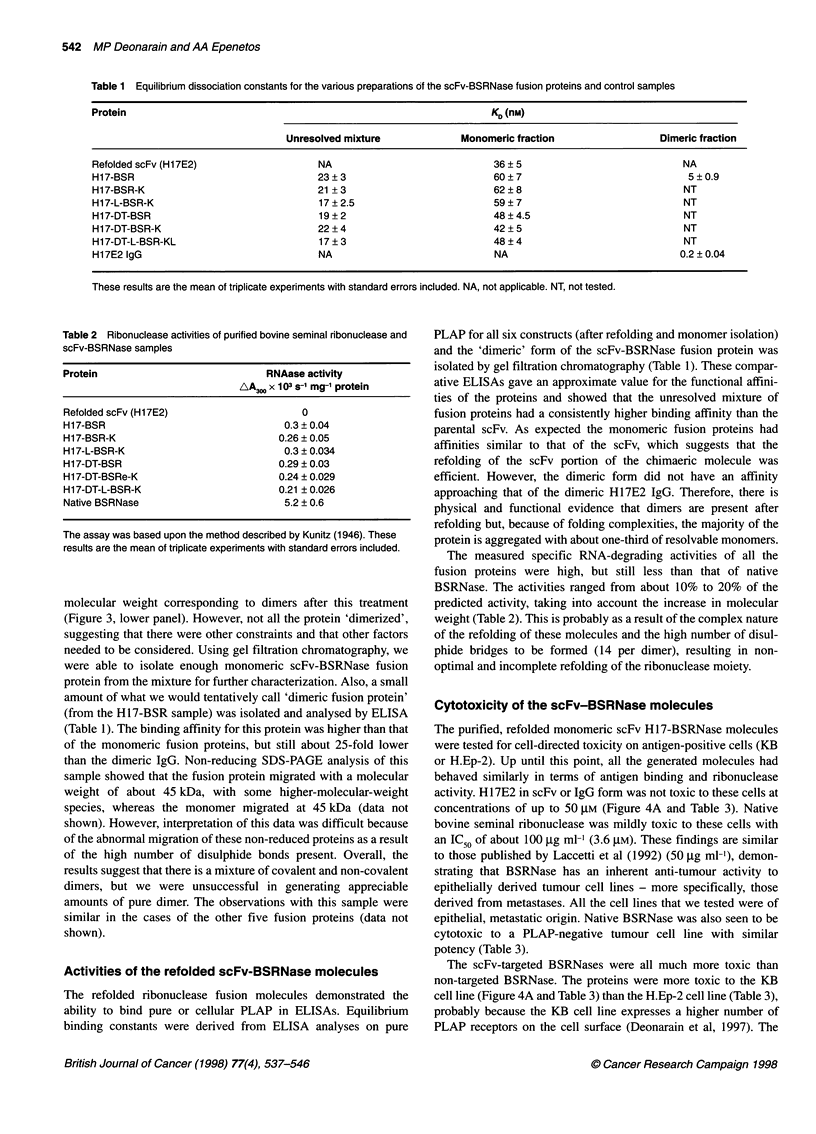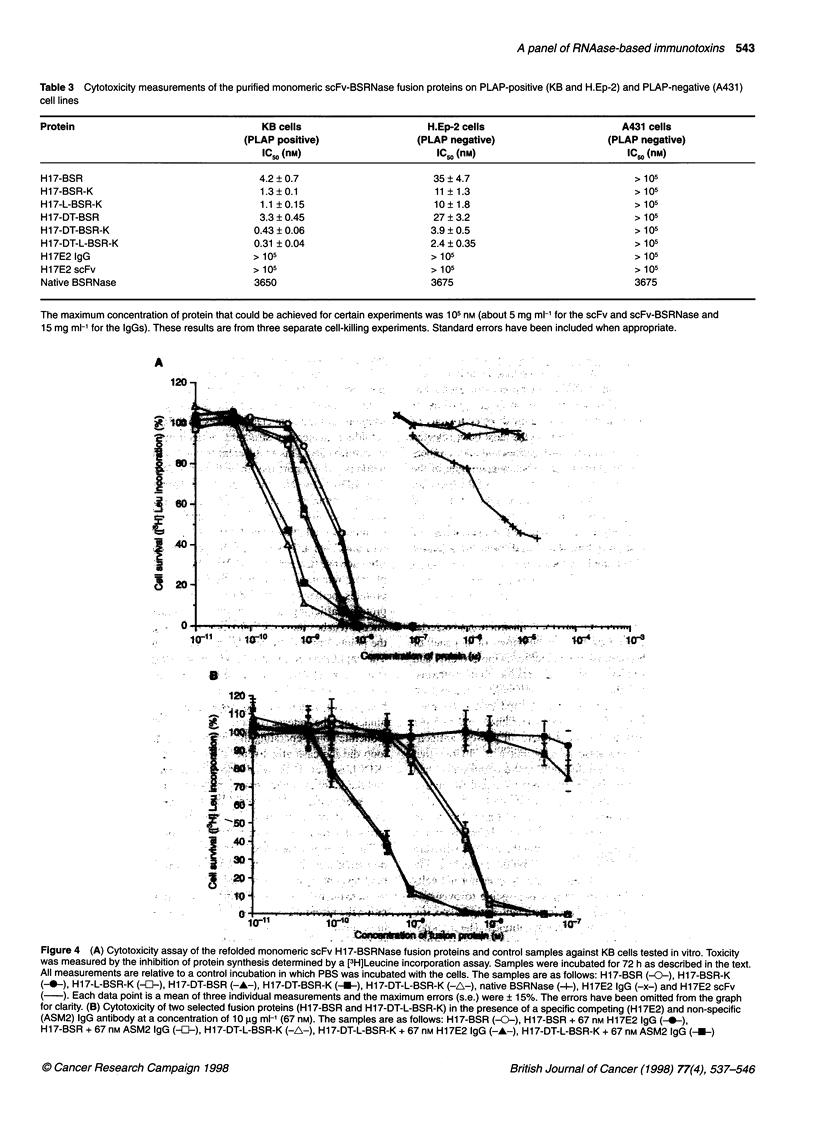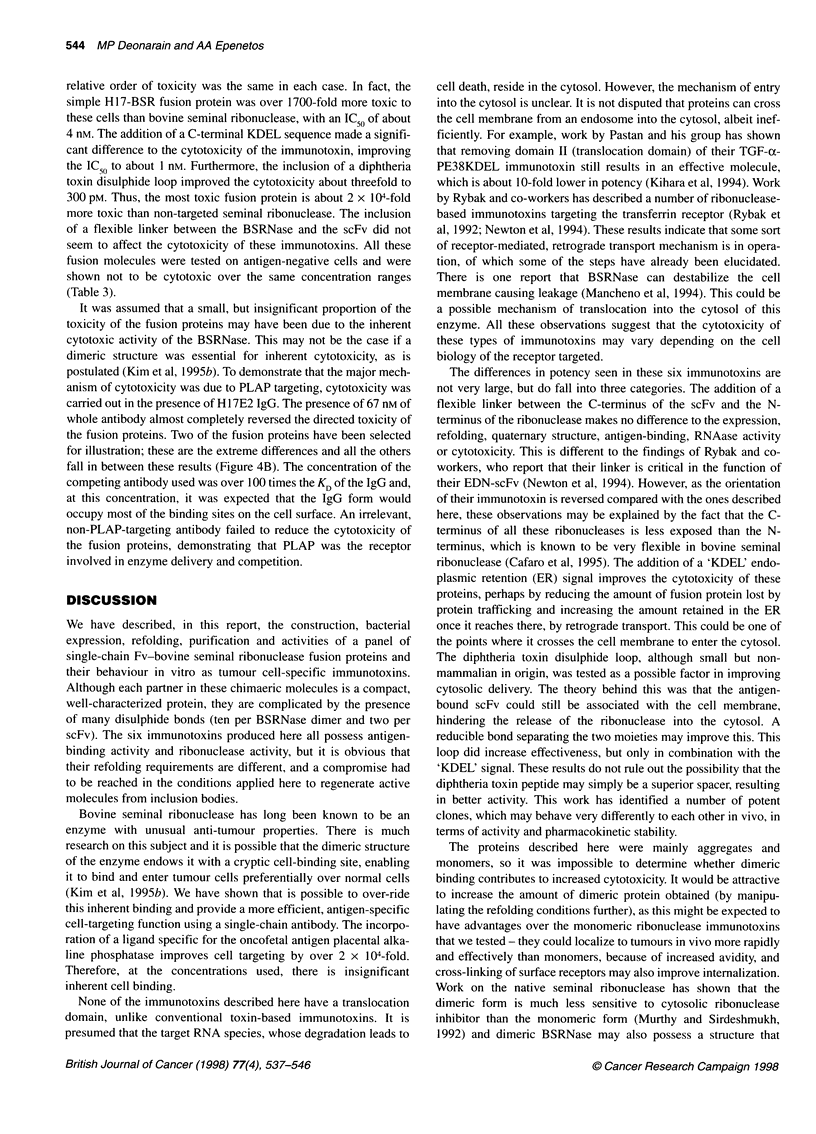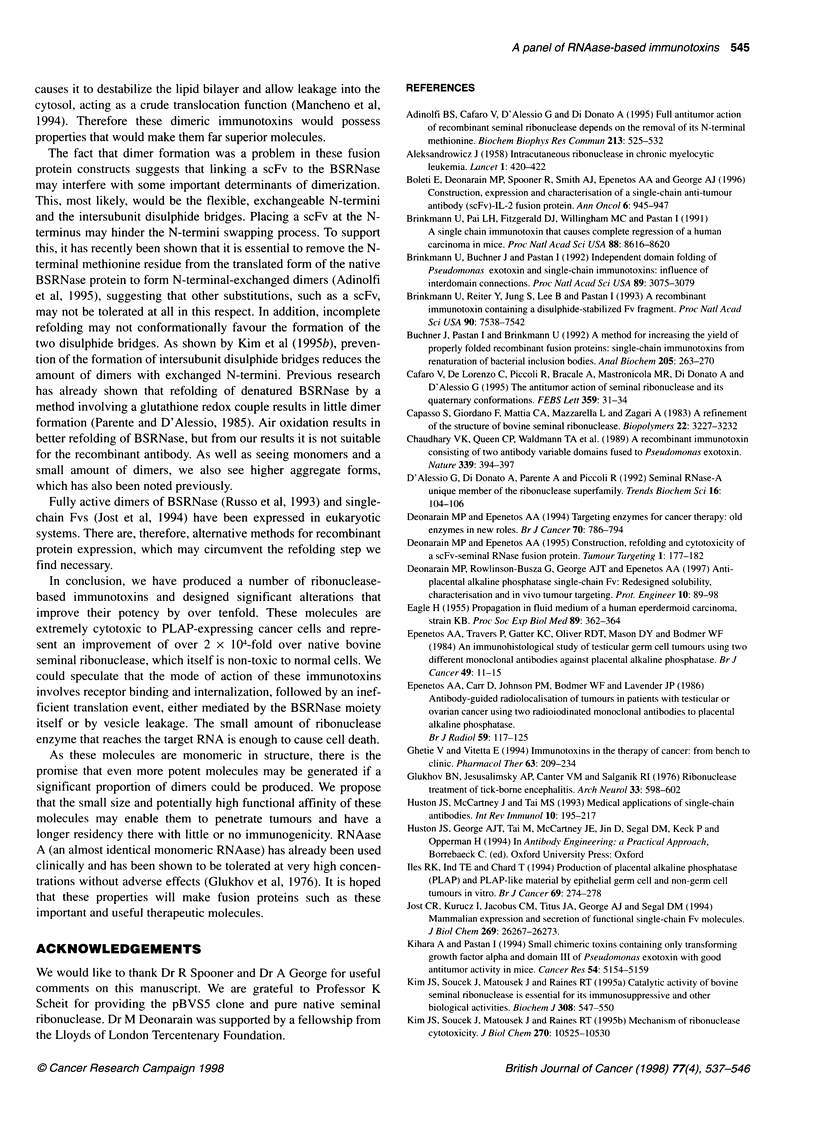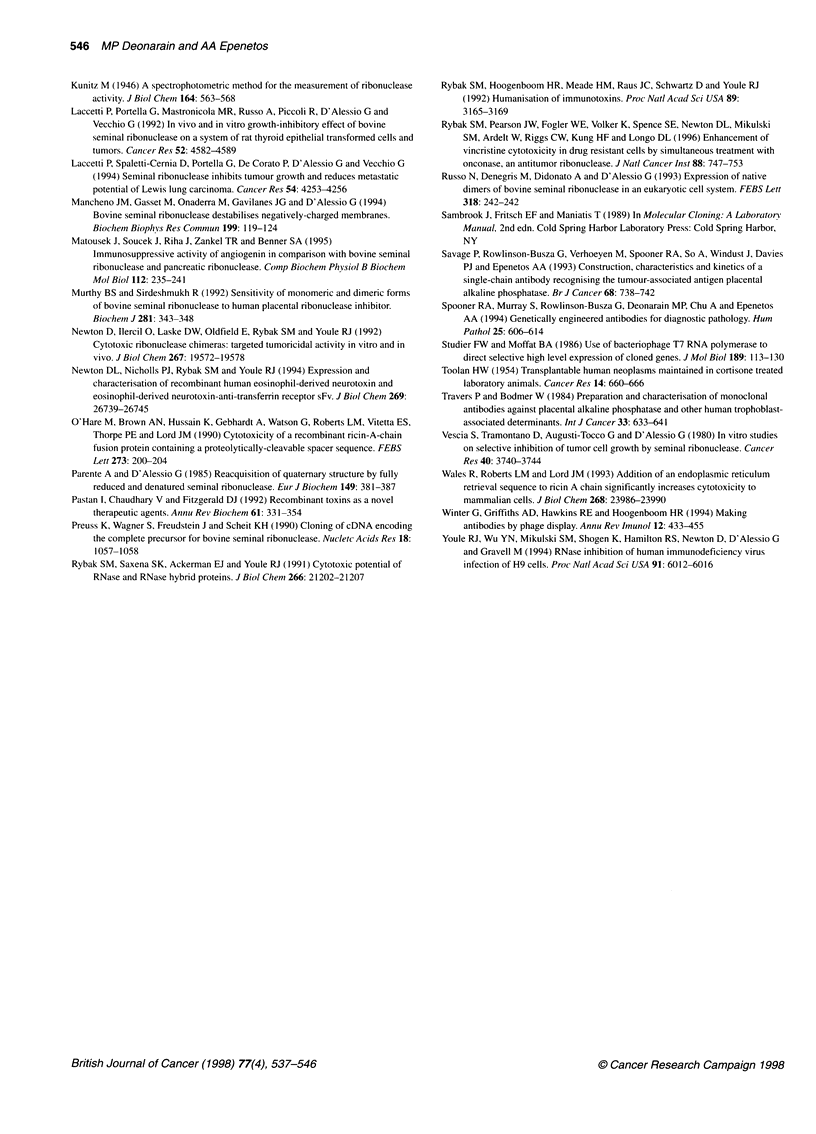# Design, characterization and anti-tumour cytotoxicity of a panel of recombinant, mammalian ribonuclease-based immunotoxins.

**DOI:** 10.1038/bjc.1998.87

**Published:** 1998-02

**Authors:** M. P. Deonarain, A. A. Epenetos

**Affiliations:** Imperial Cancer Research Fund Oncology Unit, Imperial College School of Medicine at the Hammersmith Hospital, London, UK.

## Abstract

**Images:**


British Journal of Cancer (1998) 77(4), 537–546
© 1998 Cancer Research Campaign

### Design, characterization and anti-tumour cytotoxicity of a panel of recombinant, mammalian ribonuclease-based immunotoxins

**MP Deonarain[1,2] and AA Epenetos[1*]**

[1]Imperial Cancer Research Fund Oncology Unit, Imperial College School of Medicine at the Hammersmith Hospital, Du Cane Road, London W12 0HS, UK; [2]Department of Biochemistry, Imperial College of Science, Technology & Medicine, London SW7 2AY, UK

**Summary** Bovine seminal ribonuclease (BSRNase) is an unusual member of the ribonuclease superfamily, because of its remarkable anti-tumour and immunosuppressive properties. We describe here the construction, expression, purification and characterization of a panel of six immunotoxins based upon this enzyme and show that we can increase its anti-tumour activity by over $2 \times 10^4$-fold. This is achieved by improving tumour cell targeting using a single-chain Fv (scFv) directed against the oncofetal antigen placental alkaline phosphatase. As well as the simple scFv-BSRNase fusion protein, we have constructed five other derivatives with additional peptides designed to improve folding and intracellular trafficking and delivery. We find that the molecule most cytotoxic to antigen (PLAP)-positive cells in vitro is one that contains a C-terminal 'KDEL' endoplasmic reticulum retention signal and a peptide sequence derived from diphtheria toxin. All these molecules are produced in *Escherichia coli* (*E. coli*) as insoluble inclusion bodies and require extensive in vitro processing to recover antigen binding and ribonuclease activity. Despite incomplete ribonuclease activity and quaternary assembly, these molecules are promising reagents for specific chemotherapy of cancer and are potentially less harmful and immunogenic than current immunotoxins.

**Keywords:** bovine seminal ribonuclease; placental alkaline phosphatase; single-chain Fv

Toxin proteins from bacteria, fungi and plants have been well studied in the development of cytotoxic conjugate or fusion proteins under the broad term of 'immunotoxins'. Monoclonal antibodies, their immunoreactive fragments, growth factors or cytokines have been used as vehicles to direct these toxins to tumour cells overexpressing the appropriate tumour-associated antigen. Prime examples of these include *Pseudomonas* exotoxin A, diphtheria toxin and ricin, which cause cell death by interfering with essential components of the cell protein synthesis machinery (for a review see Pastan et al, 1992). Whereas these immunotoxin molecules have produced excellent results in vitro and in animal studies, the very nature of these molecules have caused disappointing results in the clinic (reviewed in Ghetie and Vitetta, 1994). These problems are due to the side-effects and immunogenicity of the immunotoxins.

Recombinant DNA technology has led to the rational design and refinement of such immunotoxins to produce more potent, less toxic, smaller, homogeneous molecules. For example, *Pseudomonas* exotoxin A has been genetically altered to make more effective immunotoxins: the N-terminal cell binding domain has been replaced by alternative ligands (Chaudhary et al, 1989) and the C-terminal REDLK sequence has been mutated to KDEL, which is the consensus human endoplasmic retention sequence (Brinkmann et al, 1991) and which improves cell trafficking. Recombinantly produced fragments of antibodies, selected against tumour-associated antigens are very often used in the construction of immunotoxins to decrease their size and improve their pharmacokinetics, particularly as this technology has advanced so rapidly with the arrival of antibody phage display (reviewed in Winter et al, 1994). Single-chain Fv fragments (scFvs, reviewed in Huston et al, 1993) or disulphide-linked Fv fragments (dsFvs) are commonly used to target toxins to target cells (Brinkmann et al, 1991, 1993).

However, despite the use of human antibody fragments and small toxins, there is the risk of an immune response against the non-human toxin, thereby reducing its effectiveness as a result of rapid clearance of subsequent doses and adverse side-effects. A novel advance in this field was the use of enzymes of mammalian or human origin in place of the toxin. It was shown that a mammalian enzyme, bovine pancreatic ribonuclease (RNAase A) could be as cytotoxic as ricin when micro-injected directly into Xenopus oocytes (Rybak et al, 1991). This observation led to the construction of immunotoxins based upon transferrin or anti-transferrin receptor antibodies linked to RNAase A (Newton et al, 1992) and to human ribonucleases from the same family: angiogenin (Rybak et al, 1992) and EDN (eosinophil-derived neurotoxin) (Newton et al, 1994). However, even before this work, it was known that bovine seminal ribonuclease (BSRNase) had inherent anti-tumour properties, without any obvious cell-targeting moiety (Vescia et al, 1980). Another anti-tumour ribonuclease, onconase, isolated from bull frogs is believed to be similar to BSRNase and is currently under intense study as an anti-tumour drug in combination with other cancer treatment modalities (Rybak et al, 1996).

Bovine seminal ribonuclease is a member of the ribonuclease superfamily, which includes RNAase A, angiogenin and EDN.

*Received 3 March 1997*
*Revised 22 July 1997*
*Accepted 23 July 1997*

*Correspondence to:* MP Deonarain, Department of Biochemistry, Imperial College of Science, Technology & Medicine, London SW7 2AY, UK

**Present address:* Antisoma, West Africa House, Hanger Lane, Ealing, London W5 3QR, UK

These enzymes are all small, compact proteins with a molecular weight of about 13 kDa. BSRNase is the only dimeric member of this family (molecular weight 26 kDa) by virtue of two intersubunit disulphide bridges. It has a number of unusual properties related to its ribonuclease activity and dimeric structure (reviewed in D'Alessio et al, 1992; Kim et al, 1995*a* and *b*). These include anti-tumour cell activity, which is more pronounced on metastatic-derived cells. This phenomenon has been demonstrated in vitro and in vivo (Laccetti et al, 1992, 1994). In addition, it has immunosuppressive activity (Matousek et al, 1995), degrades double-stranded RNA and demonstrates allosteric regulation (D'Alessio et al, 1992). The dimeric structure, mammalian origin and immunosuppressive nature of this protein makes it an attractive candidate for an immunotoxin. It is anticipated that, although this protein is of bovine origin, it will be very well tolerated in humans, as the homologous RNAase A has been used clinically to treat tick-borne viral encephalitis (Glukhov et al, 1976) and chronic myelocytic leukaemia (Alekandrowicz, 1958), with no adverse or immunogenic reactions. The ribonuclease and cell-targeting activities of bovine seminal RNAase and onconase are being tested in anti-viral experiments to treat human immunodeficiency virus infections (Youle et al, 1994).

We describe here the construction, bacterial expression, purification and characterization of a number of scFv–BSRNase immunotoxins. The scFv is directed against the tumour-associated antigen human placental-like alkaline phosphatase (PLAP), which is an isoform of the placental alkaline phosphatase. This antigen is expressed on many tumours, such as ovarian and testicular, as well as some bladder and head and neck cancers (Epenetos et al, 1984; Iles et al, 1994). The antibody used is the well-studied H17E2 (Travers and Bodmer, 1984; Epenetos et al, 1986). The single-chain Fv has been constructed (Savage et al, 1993) and characterized in detail in vitro and in vivo (Deonarain et al, 1997). We have previously shown that this recombinant antibody localizes to tumours more rapidly than the IgG form in a mouse xenograft model of human cancer (Deonarain et al, 1997); it can be used to target interleukin 2 to tumour cells (Boleti et al, 1986) and diagnostically in immunohistochemical staining for PLAP-positive tumours (Spooner et al, 1994).

The immunotoxins described in this report differ by the addition of sequences designed to improve activity and potentiate the cytotoxicity of the molecule, based upon previous work; the addition of a disulphide-bond-forming loop from diphtheria toxin can greatly increase immunotoxin cytotoxicity (O'Hare et al, 1990) and a 'KDEL' endoplasmic reticulum retention signal can increase ricin immunotoxin cytotoxicity by ten- to 250-fold (Wales et al, 1993). In addition, it has been shown that peptides linking chimaeric molecules can modulate the activity of the molecule (Brinkmann et al, 1992). We envisage that tumour targeting of fusions of mammalian enzymes such as these could potentially improve upon conventional immunotoxins, because of their increased cytotoxicity when targeted to the correct cellular compartments, the absence of any side-effects and their expected lower or complete lack of immunogenicity in clinical use (Deonarain and Epenetos, 1994).

### MATERIALS AND METHODS

### Materials

Cell lines H.Ep-2, a human larynx epidermal carcinoma (Toolan, 1954), A431, a human eperdermoid carcinoma and all cell culture media were supplied by the cell culture services of the Imperial Cancer Research Fund (London, UK). Cell line KB, a human oral epidermal carcinoma (Eagle, 1955), was a gift from Dr Ray Iles (St Bartholomew's Hospital, London, UK). The antibody H17E2 (Travers and Bodmer, 1984) was supplied by the hybridoma unit of the Imperial Cancer Research Fund. The anti-mucin antibody ASM2 was supplied by Dr Nigel Courtenay-Luck (Antisoma). Polyclonal antibodies against the native and denatured form of bovine seminal ribonuclease for detection in ELISAs and western blotting were prepared as described in Deonarain and Epenetos (1995). The H17E2 scFv plasmid constructs and proteins have been described previously (Deonarain et al, 1997). Pure native seminal ribonuclease was a gift from Professor K Scheit (Max-Plank Institute, Heidelberg, Germany).

### Construction of the different scFv-BSRNase fusion protein genes

All recombinant techniques used were essentially as described in Sambrook et al (1989). The plasmid pBSV5, containing the gene for the secreted form of the bovine seminal ribonuclease enzyme, was kindly provided by Professor K Scheit (Preuss et al, 1990). A *XhoI*/*Eco*RI cassette containing the gene for the processed form of the enzyme was obtained by polymerase chain reaction (PCR) amplification with the following conditions: 30 cycles of 94°C (1 min), 72°C (1 min), 65°C (1 min) preceded by a 'hot start' and followed by 3 min at 72°C from this plasmid using the primers BSRNXHO 5′-GTGCGGGTCCTCGAGATCAAGCG-CAAGGAATCTGCAGCTGCC-3′ and BSRCECO 5′-GAAGC-GGTGGAATTCACTGTTCTGGCCTCAGGT-3′. The gene for the BSRNase was fused to the C-terminus of the kappa light-chain variable region of the insolubly expressed H17E2 scFv gene in plasmid pSPH17E2 (Deonarain et al, 1997) to form the plasmid pSPH17-BSR. An N-terminal linker/spacer (Ala-Pro-Ala-Ala-Ser-Pro-Ala-Asp-Ala), to separate the scFv from the BSRNase, was incorporated by amplifying the BSRNase gene with the N-terminal primer BSRLINK 5′-GTGCGGGTCCTCGAGAT-CAAGGCACCTGCTGCCTCCCCGGCAGACGCTAA GGATC-TGCAGCTGCC-3′ and the C-terminal primer BSRCECO. A C-terminal Lys-Asp-Glu-Leu sequence (KDEL) was attached by amplifying the BSRNase gene with the C-terminal primer BSRKDEL 5′-CTGGGCGTGGAATTCTTATTACAGTTCGTC-CTTCACTGAAGCATCGAA-3′ and the N-terminal primer BSRNXHO. A 22-amino-acid sequence containing the diphtheria toxin disulphide loop (O'Hare et al, 1990) was inserted between the scFv and BSRNase as a *XhoI*/*SalI* cassette obtained by PCR amplification from clone pRAS50 (R Spooner, unpublished results) using the primers DTLOOPFW 5′-GGCTGACGTCTCGAGATCAAGCGCTCTAGCCTC-GAAGGTGGGTGCGCTGGTAATAG-3′ and DTLOOPBK 5′-AATTCGGACGTCGACGTCGAGACCACCGCAAGACA-GGC-3′. Six constructs were made in total, with different permutations of N-terminal linker, C-terminal KDEL and diphtheria toxin disulphide loop. In order to facilitate purification of these proteins, six more plasmid constructs were made in which a hexahistidine affinity peptide was placed at the N-terminus, thus allowing isolation by immobilized metal affinity chromatography. The N-terminus was chosen to avoid interfering with the C-terminal 'KDEL' sequences (see below). The *pel* B leader sequence was also removed at this stage as it was not required (the proteins were not secreted solubly, see below). These alterations were

 © Cancer Research Campaign 1998

**Figure 1** (**A**) Construction of the various scFv H17–bovine seminal ribonuclease fusion genes. The parental scFv-expressing plasmid is pSPH17 (Deonarain et al, 1997) and all fusion constructs are derived from this. Each BSRNase cassette was obtained by PCR amplification of the gene from the plasmid pBSV5 (Preuss et al, 1990) using the oligonucleotides described in the text. Six plasmid constructs were made in total, each with a prefix pSP followed by 'H17' for the scFv H17E2, 'BSR' for bovine seminal ribonuclease, 'K' for the C-terminal KDEL, 'L' for the N-terminal linker and 'DT' for the diphtheria toxin disulphide loop. The N-terminal domain of the scFv was replaced at a later stage to incorporate a $His_6$ affinity purification tag and to remove the *pel* B leader sequence on all six original constructs to give the final constructs illustrated. (**B**) Schematic diagram illustrating the intended modular domain structure of the constructed immunotoxins, with different permutations of the additional sequences carried out in a single PCR reaction using primers H17HISTAG 5′-TCAGAAGCTTGCATGCAAATTCTATTTC-AAGGAGACAGTCATAATGAAACACCACCATCAC-CACCATGCCCAGCTGCAGGAG-3′ and H17SAC I 5′-GGCTG-GAGACTGGGTGAGCTCGAT to create a new N-terminal *Hin*dIII/*Sac*I cassette, which replaced the original fragment in each of the fusion protein constructs (Figure 1A).

All of the constructs were sequenced by dideoxy sequencing to ensure that no spurious alterations had arisen. The construction of these clones is illustrated in Figure 1A and for clarity the predicted structures and modular assemblies of the fusion proteins are shown schematically in Figure 1B.

### Expression of recombinant protein

*E. coli* BL21(λDE3) cells (Studier and Moffat, 1986) were transformed by the plasmids for the scFv (Deonarain et al, 1997) and fusion proteins. Single colonies were picked, re-streaked and grown overnight at 37°C in L-Broth supplemented with 100 μg ml$^{-1}$ ampicillin. These starter cultures were used to inoculate 500-ml cultures and were grown to an $A_{600}$ of 1.0. At this point, IPTG was added to 0.5 mM and induction of recombinant protein was allowed for 3–6 h at 37°C. Cell pellets were harvested by centrifugation and stored at –70°C until required.

### Refolding and purification of recombinant protein

The expressed proteins accumulated as cytoplasmic inclusion bodies, which were recovered from the insoluble cell components as described by Buchner et al (1992) with slight modification: spheroplasts were prepared from the cell pellets by osmotic shock in 20% sucrose. The spheroplasts were resuspended (40 ml l$^{-1}$ of cell culture) in buffer A (50 mM Tris-HCl, 20 mM EDTA, pH 8.0), lysozyme was added to 200 μg ml$^{-1}$, and the suspension was mixed by inversion for 2–16 h at 4°C. The suspension was then sonicated on ice, Triton X-100 was added to 2% and sodium chloride was added to 0.5 M. This mixture was left to incubate at room temperature for 30 min. The lysate was centrifuged at 25 000 r.p.m. in a

© Cancer Research Campaign 1998

Beckman 45Ti rotor at 10°C for 30 min. The insoluble material was resuspended in 40 ml of buffer A, sonicated on ice and centrifuged as above. This process was repeated a total of four times until semi-pure, washed inclusion bodies were isolated. The final centrifugation step was used to aliquot the samples in batches for storage at –70°C. Initial attempts to obtain pure protein were unsuccessful until hexahistidine affinity tags were re-engineered onto all of the clones. These inclusion bodies were further purified as follows: the insoluble pellet was dissolved (10 ml $l^{-1}$ of original culture) in 6 M guanidine hydrogen chloride (GuHCl), 50 mM Tris-HCl, 5 mM magnesium chloride, pH 8.0, for 2–16 h. Undissolved material was removed by ultracentrifugation at 30 000 r.p.m. for 30 min at 15°C. The clarified, denatured inclusion body preparation was applied to a 5-ml chelating sepharose (Fast-flow, Pharmacia) column, charged with copper (II) ions and equilibrated in buffer B (8 M urea, 50 mM Tris-HCl, pH 8.0). The protein was allowed to bind by gentle inversion for 2–16 h at 4°C. The column was then packed down, washed and eluted as follows: 50 ml of buffer B, 50 ml each of 10 mM, 20 mM, 40 mM and 150 mM imidazole dissolved in buffer B. The column was finally stripped by washing with 50 ml of 50 mM EDTA, 0.5 M sodium chloride. The majority of pure scFv–BSRNase protein eluted at 150 mM imidazole. The pure fractions (as judged by SDS-PAGE) were pooled, concentrated to about 5 mg $ml^{-1}$ in a volume of 2–5 ml and reduced by the addition of dithioerythritol to a final concentration of 0.3 M for 1 h. This process yielded pure, reduced and denatured fusion protein.

Refolding was carried out by rapid dilution of the reduced and denatured protein, 1:100 in a refolding buffer containing 0.5 M L-arginine, 100 mM Tris-HCl, 2 mM EDTA and 4 mM oxidized glutathione (GSSG), pH 8.0. This was carried out at 10°C and the samples were incubated at 10°C for 48–72 h. Refolded samples (0.5–1.0 l) were dialysed against 20 mM Tris-HCl, pH 8.0 (two changes of 5–10 l each), concentrated 50-fold by ultrafiltration in an Amicon 8400 stirrer cell with a PM30 membrane and dialysed exhaustively against 20 mM Tris-HCl, pH 8.0. In order to promote the formation of disulphide-linked dimers, the refolded samples were reduced by the addition of DTT to 2 mM and incubated for 1 h. These samples were then dialysed as before to remove excess DTT and glutathione. Samples were dialysed into phosphate-buffered saline (PBS) for cell binding and cytotoxicity studies or stored frozen until required.

### Enzyme-linked immunosorbent assays and Western blots

ELISAs were carried out in 96-well microtitre plates coated in 20 μg $ml^{-1}$ hPLAP in PBS or $10^5$ cells per well KB or H.Ep-2 cells, fixed with 0.25% gluteraldehyde (as described in Deonarain and Epenetos, 1995). The bound samples were detected using rabbit anti-sera against pure native seminal ribonuclease followed by donkey anti-rabbit horseradish peroxidase diluted in PBST (PBS with 0.05% Tween-20).

For Western blot analysis, proteins were separated by 15% SDS-PAGE under reducing conditions as described in Deonarain and Epenetos (1995) and transferred onto nitrocellulose using a Biorad semi-dry blotting apparatus, under the manufacturer's conditions. The membranes were blocked with 5% non-fat milk protein in PBST and proteins detected as described for ELISA using polyclonal antibodies against the denatured form of the enzyme.

### Analytical and preparative gel filtration

Analyses of the native molecular weights and quaternary structures of the fusion proteins were carried out by gel filtration on a Superose-6 column (Pharmacia), equilibrated in 20 mM Tris-HCl, pH 8.0, 0.1 M sodium chloride. Samples eluting at volumes corresponding to monomers (45 kDa) and dimers (90 kDa) were collected and characterized.

### Ribonuclease assays

Qualitative RNA-degrading assays were carried out by incubating 5 μg of yeast RNA with the sample and analysing on an agarose gel, as described before (Deonarain and Epenetos, 1995). Quantitative assays were performed by spectrophotometrically following the decrease in $A_{300}$ of the RNA in a 1-cm pathlength cuvette containing 1 mg $ml^{-1}$ yeast RNA, 0.1 M Tris-HCl, 0.15 M sodium chloride, pH 7.5, at room temperature (based on an assay described in Kunitz (1946)). These measurements were made with reference to standard blank and native seminal RNAase-containing samples.

### Cytoxicity assays

The refolded proteins were dialysed against PBS and tested for cell-killing activity on antigen-positive cell lines KB and H.Ep-2 and on the antigen-negative cell line A431. In each assay, $10^4$ cells were grown in a 96-well microtitre plate in a volume of 100 μl of media (2% RPMI 1640/10% fetal calf serum). Then, 20 μl, appropriately diluted in PBS and 0.2-μm filtered, of refolded fusion protein were added to each well in triplicate. The samples were incubated at 37°C/5% carbon dioxide for 72 h. Cell viability was determined by measuring the level of protein synthesis in a [$^3$H] Leucine incorporation assay. In this assay, 1 μCi of [$^3$H] Leu was added per well and incubated for 4 h at 37°C/5% carbon dioxide. The cells were harvested with LKB betaplate harvester and the cellular incorporated radioactivity was counted. Control samples used were PBS, native seminal ribonuclease, whole IgG and scFv H17E2. For competition conditions, the above assay was carried out in the presence of 10 μg $ml^{-1}$ (67 nM) H17E2 IgG and ASM2 IgG.

**Figure 2** Expression (left panel) of all six scFv H17E2-BSRNase fusion proteins in *E. coli* strain BL21 (λDE3) and their purification (right panel). Lane 1, scFv H17E2; lane 2, H17-BSR; lane 3, H17-BSR-K; lane 4, H17-L-BSR-K; lane 5, H17-DT-BSR; lane 6, H17-DT-BSR-K; lane 7, H17-DT-L-BSR-K; M, molecular weight markers. Proteins were analysed on a 10% reducing and denaturing polyacrylamide gel stained with Comassie Brilliant Blue R-250

© Cancer Research Campaign 1998

### RESULTS

### Construction and expression of the scFv-BSRNase fusion proteins

The initial scFv-BSRNase fusion protein construct consisted of a straightforward linkage of the C-terminus of the scFv $V_\kappa$ region to the native BSRNase N-terminus. This site was chosen as examination of the three-dimensional structure indicated that the C-terminus was less accessible (Capasso et al, 1983). The amplification oligonucleotides were designed to produce a BSRNase cassette with an N-terminal *Xho*I site followed by a Ile-Lys-Arg sequence to replace the same sequence found at the end of the scFv (Figure 1A). Further constructs were made with a C-terminal 'Lys-Asp-Glu-Leu' sequence (KDEL), which was introduced by PCR amplification. Another strategy designed to improve the immunotoxin potency was the addition of a peptide sequence from diphtheria toxin, which was predicted to form a disulphide-containing loop. It was hoped that the addition of this sequence would allow the release of the ribonuclease portion of the immunotoxin from the antigen-bound scFv, once inside the reducing environment of the cytosol. The final modification of the scFv-BSRNase fusion protein constructs was the inclusion of a spacer/linker sequence between the scFv and the BSRNase. This was added to separate further the two portions of the molecule to allow better independent folding (as observed for *Pseudomonas* exotoxin immunotoxins; Brinkmann et al, 1992). In addition, it has been shown that the N-termini of the BSRNase dimer are exchanged across the subunit interface in some forms of the dimer (Cafaro et al, 1995). Thus, a flexible linker between the two moieties may reduce steric hindrance that may interfere with this exchange process.

Initially, the expressed fusion proteins were found to be mostly cytoplasmic inclusion bodies with a little insoluble periplasmic material, despite the inclusion of a *pel* B leader sequence. This was not surprising, as the parental scFv is expressed insolubly (Deonarain et al, 1997) and many bacterially expressed ribonucleases are insoluble. Expression levels were high, with about 10% of the total cellular protein being recombinant protein. The expressed proteins were analysed by SDS-PAGE under reducing conditions and found to have approximately the expected molecular weight.

Initial experiments indicated that the *pel* B leader sequence was not required, therefore a second set of constructs was made to incorporate a $His_6$ affinity purification tag at the N-terminus, in place of the leader. This was done so as not to interfere with the C-termini of the ribonuclease enzyme, some of which possessed the KDEL sequence. This latter sequence must be at the extreme C-terminus to function effectively. These new constructs were made by replacing the same *Hin*dIII/*Sac*I cassette with a PCR-mutated version containing the alterations (Figure 1A). Their expression in *E. coli* BL21 (λDE3) was similar to that of the unaltered forms and they were of, approximately, the expected molecular weight (Figure 2, left panel).

### Purification and refolding of the recombinant proteins

Attempts to purify the initially constructed scFv-BSRNase fusion proteins (without $His_6$ tags) were unsuccessful, as immobilized placental alkaline phosphatase was not stable under the purification conditions tested. In contrast, immobilized metal ion affinity chromatography of the $His_6$-containing proteins under denaturing conditions yielded essentially pure protein for all six constructs (Figure 2, right panel). Each protein had approximately the correct predicted molecular weight and were the correct relative size (predicted weight in daltons): H17-BSR, 45 024; H17-BSR-K, 45 450; H17-L-BSR-K, 46 223; H17-DT-BSR, 46 444; H17-DT-BSR-K, 46 800; H17-DT-L-BSR-K, 47 222.

Many methods, which have already been described, have been tested for refolding the initial scFv–BSRNase fusion protein (Deonarain and Epenetos, 1995). Each of these methods varied in their efficiency to completely regenerate active chimaeric molecules. The method eventually used was based on that of Buchner et al (1992), which gave good antigen-binding activity as well as reasonable but not complete ribonuclease activity (see below), and was chosen as an acceptable compromise.

### Molecular weights of the fusion proteins

Gel filtration chromatography analyses of freshly refolded H17-BSR fusion protein showed that there was a mixture (approximately 1:2) of monomeric (45 kDa) and aggregated (>250 kDa) proteins, indicating that the refolding method was not effective in regenerating dimers of seminal ribonuclease (Figure 3, upper panel). We hypothesized that the intersubunit disulphide bonds were blocked by the glutathione used in the refolding mixture, thus preventing association of the protomers. Reduction with DTT followed by exhaustive dialysis seemed to confirm this, as a proportion of the fusion protein was formed into species with a

**Figure 3** Gel filtration traces of the purified fusion proteins after refolding, before (upper figure) and after (lower figure) reduction/dialysis treatment to promote dimer formation. A indicates the position of higher-molecular-weight aggregates, D indicates the expected position of dimers and M indicates the expected position of monomers

© Cancer Research Campaign 1998

**Table 1** Equilibrium dissociation constants for the various preparations of the scFv-BSRNase fusion proteins and control samples

| Protein | $K_D$ (nM) | | |
|---|---|---|---|
| | Unresolved mixture | Monomeric fraction | Dimeric fraction |
| Refolded scFv (H17E2) | NA | 36 ± 5 | NA |
| H17-BSR | 23 ± 3 | 60 ± 7 | 5 ± 0.9 |
| H17-BSR-K | 21 ± 3 | 62 ± 8 | NT |
| H17-L-BSR-K | 17 ± 2.5 | 59 ± 7 | NT |
| H17-DT-BSR | 19 ± 2 | 48 ± 4.5 | NT |
| H17-DT-BSR-K | 22 ± 4 | 42 ± 5 | NT |
| H17-DT-L-BSR-KL | 17 ± 3 | 48 ± 4 | NT |
| H17E2 IgG | NA | NA | 0.2 ± 0.04 |

These results are the mean of triplicate experiments with standard errors included. NA, not applicable. NT, not tested.

**Table 2** Ribonuclease activities of purified bovine seminal ribonuclease and scFv-BSRNase samples

| Protein | RNAase activity $\triangle A_{300} \times 10^3$ s$^{-1}$ mg$^{-1}$ protein |
|---|---|
| Refolded scFv (H17E2) | 0 |
| H17-BSR | 0.3 ± 0.04 |
| H17-BSR-K | 0.26 ± 0.05 |
| H17-L-BSR-K | 0.3 ± 0.034 |
| H17-DT-BSR | 0.29 ± 0.03 |
| H17-DT-BSRe-K | 0.24 ± 0.029 |
| H17-DT-L-BSR-K | 0.21 ± 0.026 |
| Native BSRNase | 5.2 ± 0.6 |

The assay was based upon the method described by Kunitz (1946). These results are the mean of triplicate experiments with standard errors included.

molecular weight corresponding to dimers after this treatment (Figure 3, lower panel). However, not all the protein 'dimerized', suggesting that there were other constraints and that other factors needed to be considered. Using gel filtration chromatography, we were able to isolate enough monomeric scFv-BSRNase fusion protein from the mixture for further characterization. Also, a small amount of what we would tentatively call 'dimeric fusion protein' (from the H17-BSR sample) was isolated and analysed by ELISA (Table 1). The binding affinity for this protein was higher than that of the monomeric fusion proteins, but still about 25-fold lower than the dimeric IgG. Non-reducing SDS-PAGE analysis of this sample showed that the fusion protein migrated with a molecular weight of about 45 kDa, with some higher-molecular-weight species, whereas the monomer migrated at 45 kDa (data not shown). However, interpretation of this data was difficult because of the abnormal migration of these non-reduced proteins as a result of the high number of disulphide bonds present. Overall, the results suggest that there is a mixture of covalent and non-covalent dimers, but we were unsuccessful in generating appreciable amounts of pure dimer. The observations with this sample were similar in the cases of the other five fusion proteins (data not shown).

### Activities of the refolded scFv-BSRNase molecules

The refolded ribonuclease fusion molecules demonstrated the ability to bind pure or cellular PLAP in ELISAs. Equilibrium binding constants were derived from ELISA analyses on pure PLAP for all six constructs (after refolding and monomer isolation) and the 'dimeric' form of the scFv-BSRNase fusion protein was isolated by gel filtration chromatography (Table 1). These comparative ELISAs gave an approximate value for the functional affinities of the proteins and showed that the unresolved mixture of fusion proteins had a consistently higher binding affinity than the parental scFv. As expected the monomeric fusion proteins had affinities similar to that of the scFv, which suggests that the refolding of the scFv portion of the chimaeric molecule was efficient. However, the dimeric form did not have an affinity approaching that of the dimeric H17E2 IgG. Therefore, there is physical and functional evidence that dimers are present after refolding but, because of folding complexities, the majority of the protein is aggregated with about one-third of resolvable monomers.

The measured specific RNA-degrading activities of all the fusion proteins were high, but still less than that of native BSRNase. The activities ranged from about 10% to 20% of the predicted activity, taking into account the increase in molecular weight (Table 2). This is probably as a result of the complex nature of the refolding of these molecules and the high number of disulphide bridges to be formed (14 per dimer), resulting in non-optimal and incomplete refolding of the ribonuclease moiety.

### Cytotoxicity of the scFv–BSRNase molecules

The purified, refolded monomeric scFv H17-BSRNase molecules were tested for cell-directed toxicity on antigen-positive cells (KB or H.Ep-2). Up until this point, all the generated molecules had behaved similarly in terms of antigen binding and ribonuclease activity. H17E2 in scFv or IgG form was not toxic to these cells at concentrations of up to 50 μM (Figure 4A and Table 3). Native bovine seminal ribonuclease was mildly toxic to these cells with an $IC_{50}$ of about 100 μg ml$^{-1}$ (3.6 μM). These findings are similar to those published by Laccetti et al (1992) (50 μg ml$^{-1}$), demonstrating that BSRNase has an inherent anti-tumour activity to epithelially derived tumour cell lines – more specifically, those derived from metastases. All the cell lines that we tested were of epithelial, metastatic origin. Native BSRNase was also seen to be cytotoxic to a PLAP-negative tumour cell line with similar potency (Table 3).

The scFv-targeted BSRNases were all much more toxic than non-targeted BSRNase. The proteins were more toxic to the KB cell line (Figure 4A and Table 3) than the H.Ep-2 cell line (Table 3), probably because the KB cell line expresses a higher number of PLAP receptors on the cell surface (Deonarain et al, 1997). The

© Cancer Research Campaign 1998

**Table 3** Cytotoxicity measurements of the purified monomeric scFv-BSRNase fusion proteins on PLAP-positive (KB and H.Ep-2) and PLAP-negative (A431) cell lines

| Protein | KB cells (PLAP positive) $IC_{50}$ (nM) | H.Ep-2 cells (PLAP negative) $IC_{50}$ (nM) | A431 cells (PLAP negative) $IC_{50}$ (nM) |
|---|---|---|---|
| H17-BSR | 4.2 ± 0.7 | 35 ± 4.7 | > $10^5$ |
| H17-BSR-K | 1.3 ± 0.1 | 11 ± 1.3 | > $10^5$ |
| H17-L-BSR-K | 1.1 ± 0.15 | 10 ± 1.8 | > $10^5$ |
| H17-DT-BSR | 3.3 ± 0.45 | 27 ± 3.2 | > $10^5$ |
| H17-DT-BSR-K | 0.43 ± 0.06 | 3.9 ± 0.5 | > $10^5$ |
| H17-DT-L-BSR-K | 0.31 ± 0.04 | 2.4 ± 0.35 | > $10^5$ |
| H17E2 IgG | > $10^5$ | > $10^5$ | > $10^5$ |
| H17E2 scFv | > $10^5$ | > $10^5$ | > $10^5$ |
| Native BSRNase | 3650 | 3675 | 3675 |

The maximum concentration of protein that could be achieved for certain experiments was $10^5$ nM (about 5 mg $ml^{-1}$ for the scFv and scFv-BSRNase and 15 mg $ml^{-1}$ for the IgGs). These results are from three separate cell-killing experiments. Standard errors have been included when appropriate.

**Figure 4** (**A**) Cytotoxicity assay of the refolded monomeric scFv H17-BSRNase fusion proteins and control samples against KB cells tested in vitro. Toxicity was measured by the inhibition of protein synthesis determined by a [$^3$H]Leucine incorporation assay. Samples were incubated for 72 h as described in the text. All measurements are relative to a control incubation in which PBS was incubated with the cells. The samples are as follows: H17-BSR (–○–), H17-BSR-K (–●–), H17-L-BSR-K (–□–), H17-DT-BSR (–▲–), H17-DT-BSR-K (–■–), H17-DT-L-BSR-K (–△–), native BSRNase (–+–), H17E2 IgG (–x–) and H17E2 scFv (——). Each data point is a mean of three individual measurements and the maximum errors (s.e.) were ± 15%. The errors have been omitted from the graph for clarity. (**B**) Cytotoxicity of two selected fusion proteins (H17-BSR and H17-DT-L-BSR-K) in the presence of a specific competing (H17E2) and non-specific (ASM2) IgG antibody at a concentration of 10 μg $ml^{-1}$ (67 nM). The samples are as follows: H17-BSR (–○–), H17-BSR + 67 nM H17E2 IgG (–●–), H17-BSR + 67 nM ASM2 IgG (–□–), H17-DT-L-BSR-K (–△–), H17-DT-L-BSR-K + 67 nM H17E2 IgG (–▲–), H17-DT-L-BSR-K + 67 nM ASM2 IgG (–■–)

© Cancer Research Campaign 1998

relative order of toxicity was the same in each case. In fact, the simple H17-BSR fusion protein was over 1700-fold more toxic to these cells than bovine seminal ribonuclease, with an $IC_{50}$ of about 4 nM. The addition of a C-terminal KDEL sequence made a significant difference to the cytotoxicity of the immunotoxin, improving the $IC_{50}$ to about 1 nM. Furthermore, the inclusion of a diphtheria toxin disulphide loop improved the cytotoxicity about threefold to 300 pM. Thus, the most toxic fusion protein is about $2 \times 10^4$-fold more toxic than non-targeted seminal ribonuclease. The inclusion of a flexible linker between the BSRNase and the scFv did not seem to affect the cytotoxicity of these immunotoxins. All these fusion molecules were tested on antigen-negative cells and were shown not to be cytotoxic over the same concentration ranges (Table 3).

It was assumed that a small, but insignificant proportion of the toxicity of the fusion proteins may have been due to the inherent cytotoxic activity of the BSRNase. This may not be the case if a dimeric structure was essential for inherent cytotoxicity, as is postulated (Kim et al, 1995*b*). To demonstrate that the major mechanism of cytotoxicity was due to PLAP targeting, cytotoxicity was carried out in the presence of H17E2 IgG. The presence of 67 nM of whole antibody almost completely reversed the directed toxicity of the fusion proteins. Two of the fusion proteins have been selected for illustration; these are the extreme differences and all the others fall in between these results (Figure 4B). The concentration of the competing antibody used was over 100 times the $K_D$ of the IgG and, at this concentration, it was expected that the IgG form would occupy most of the binding sites on the cell surface. An irrelevant, non-PLAP-targeting antibody failed to reduce the cytotoxicity of the fusion proteins, demonstrating that PLAP was the receptor involved in enzyme delivery and competition.

### DISCUSSION

We have described, in this report, the construction, bacterial expression, refolding, purification and activities of a panel of single-chain Fv–bovine seminal ribonuclease fusion proteins and their behaviour in vitro as tumour cell-specific immunotoxins. Although each partner in these chimaeric molecules is a compact, well-characterized protein, they are complicated by the presence of many disulphide bonds (ten per BSRNase dimer and two per scFv). The six immunotoxins produced here all possess antigen-binding activity and ribonuclease activity, but it is obvious that their refolding requirements are different, and a compromise had to be reached in the conditions applied here to regenerate active molecules from inclusion bodies.

Bovine seminal ribonuclease has long been known to be an enzyme with unusual anti-tumour properties. There is much research on this subject and it is possible that the dimeric structure of the enzyme endows it with a cryptic cell-binding site, enabling it to bind and enter tumour cells preferentially over normal cells (Kim et al, 1995*b*). We have shown that is possible to over-ride this inherent binding and provide a more efficient, antigen-specific cell-targeting function using a single-chain antibody. The incorporation of a ligand specific for the oncofetal antigen placental alkaline phosphatase improves cell targeting by over $2 \times 10^4$-fold. Therefore, at the concentrations used, there is insignificant inherent cell binding.

None of the immunotoxins described here have a translocation domain, unlike conventional toxin-based immunotoxins. It is presumed that the target RNA species, whose degradation leads to cell death, reside in the cytosol. However, the mechanism of entry into the cytosol is unclear. It is not disputed that proteins can cross the cell membrane from an endosome into the cytosol, albeit inefficiently. For example, work by Pastan and his group has shown that removing domain II (translocation domain) of their TGF-α-PE38KDEL immunotoxin still results in an effective molecule, which is about 10-fold lower in potency (Kihara et al, 1994). Work by Rybak and co-workers has described a number of ribonuclease-based immunotoxins targeting the transferrin receptor (Rybak et al, 1992; Newton et al, 1994). These results indicate that some sort of receptor-mediated, retrograde transport mechanism is in operation, of which some of the steps have already been elucidated. There is one report that BSRNase can destabilize the cell membrane causing leakage (Mancheno et al, 1994). This could be a possible mechanism of translocation into the cytosol of this enzyme. All these observations suggest that the cytotoxicity of these types of immunotoxins may vary depending on the cell biology of the receptor targeted.

The differences in potency seen in these six immunotoxins are not very large, but do fall into three categories. The addition of a flexible linker between the C-terminus of the scFv and the N-terminus of the ribonuclease makes no difference to the expression, refolding, quaternary structure, antigen-binding, RNAase activity or cytotoxicity. This is different to the findings of Rybak and co-workers, who report that their linker is critical in the function of their EDN-scFv (Newton et al, 1994). However, as the orientation of their immunotoxin is reversed compared with the ones described here, these observations may be explained by the fact that the C-terminus of all these ribonucleases is less exposed than the N-terminus, which is known to be very flexible in bovine seminal ribonuclease (Cafaro et al, 1995). The addition of a 'KDEL' endoplasmic retention (ER) signal improves the cytotoxicity of these proteins, perhaps by reducing the amount of fusion protein lost by protein trafficking and increasing the amount retained in the ER once it reaches there, by retrograde transport. This could be one of the points where it crosses the cell membrane to enter the cytosol. The diphtheria toxin disulphide loop, although small but non-mammalian in origin, was tested as a possible factor in improving cytosolic delivery. The theory behind this was that the antigen-bound scFv could still be associated with the cell membrane, hindering the release of the ribonuclease into the cytosol. A reducible bond separating the two moieties may improve this. This loop did increase effectiveness, but only in combination with the 'KDEL' signal. These results do not rule out the possibility that the diphtheria toxin peptide may simply be a superior spacer, resulting in better activity. This work has identified a number of potent clones, which may behave very differently to each other in vivo, in terms of activity and pharmacokinetic stability.

The proteins described here were mainly aggregates and monomers, so it was impossible to determine whether dimeric binding contributes to increased cytotoxicity. It would be attractive to increase the amount of dimeric protein obtained (by manipulating the refolding conditions further), as this might be expected to have advantages over the monomeric ribonuclease immunotoxins that we tested – they could localize to tumours in vivo more rapidly and effectively than monomers, because of increased avidity, and cross-linking of surface receptors may also improve internalization. Work on the native seminal ribonuclease has shown that the dimeric form is much less sensitive to cytosolic ribonuclease inhibitor than the monomeric form (Murthy and Sirdeshmukh, 1992) and dimeric BSRNase may also possess a structure that

 © Cancer Research Campaign 1998

causes it to destabilize the lipid bilayer and allow leakage into the cytosol, acting as a crude translocation function (Mancheno et al, 1994). Therefore these dimeric immunotoxins would possess properties that would make them far superior molecules.

The fact that dimer formation was a problem in these fusion protein constructs suggests that linking a scFv to the BSRNase may interfere with some important determinants of dimerization. This, most likely, would be the flexible, exchangeable N-termini and the intersubunit disulphide bridges. Placing a scFv at the N-terminus may hinder the N-termini swapping process. To support this, it has recently been shown that it is essential to remove the N-terminal methionine residue from the translated form of the native BSRNase protein to form N-terminal-exchanged dimers (Adinolfi et al, 1995), suggesting that other substitutions, such as a scFv, may not be tolerated at all in this respect. In addition, incomplete refolding may not conformationally favour the formation of the two disulphide bridges. As shown by Kim et al (1995*b*), prevention of the formation of intersubunit disulphide bridges reduces the amount of dimers with exchanged N-termini. Previous research has already shown that refolding of denatured BSRNase by a method involving a glutathione redox couple results in little dimer formation (Parente and D'Alessio, 1985). Air oxidation results in better refolding of BSRNase, but from our results it is not suitable for the recombinant antibody. As well as seeing monomers and a small amount of dimers, we also see higher aggregate forms, which has also been noted previously.

Fully active dimers of BSRNase (Russo et al, 1993) and single-chain Fvs (Jost et al, 1994) have been expressed in eukaryotic systems. There are, therefore, alternative methods for recombinant protein expression, which may circumvent the refolding step we find necessary.

In conclusion, we have produced a number of ribonuclease-based immunotoxins and designed significant alterations that improve their potency by over tenfold. These molecules are extremely cytotoxic to PLAP-expressing cancer cells and represent an improvement of over $2 \times 10^4$-fold over native bovine seminal ribonuclease, which itself is non-toxic to normal cells. We could speculate that the mode of action of these immunotoxins involves receptor binding and internalization, followed by an inefficient translation event, either mediated by the BSRNase moiety itself or by vesicle leakage. The small amount of ribonuclease enzyme that reaches the target RNA is enough to cause cell death.

As these molecules are monomeric in structure, there is the promise that even more potent molecules may be generated if a significant proportion of dimers could be produced. We propose that the small size and potentially high functional affinity of these molecules may enable them to penetrate tumours and have a longer residency there with little or no immunogenicity. RNAase A (an almost identical monomeric RNAase) has already been used clinically and has been shown to be tolerated at very high concentrations without adverse effects (Glukhov et al, 1976). It is hoped that these properties will make fusion proteins such as these important and useful therapeutic molecules.

### ACKNOWLEDGEMENTS

We would like to thank Dr R Spooner and Dr A George for useful comments on this manuscript. We are grateful to Professor K Scheit for providing the pBVS5 clone and pure native seminal ribonuclease. Dr M Deonarain was supported by a fellowship from the Lloyds of London Tercentenary Foundation.

### REFERENCES

Adinolfi BS, Cafaro V, D'Alessio G and Di Donato A (1995) Full antitumor action of recombinant seminal ribonuclease depends on the removal of its N-terminal methionine. *Biochem Biophys Res Commun* **213**: 525–532

Aleksandrowicz J (1958) Intracutaneous ribonuclease in chronic myelocytic leukemia. *Lancet* **1**: 420–422

Boleti E, Deonarain MP, Spooner R, Smith AJ, Epenetos AA and George AJ (1996) Construction, expression and characterisation of a single-chain anti-tumour antibody (scFv)-IL-2 fusion protein. *Ann Oncol* **6**: 945–947

Brinkmann U, Pai LH, Fitzgerald DJ, Willingham MC and Pastan I (1991) A single chain immunotoxin that causes complete regression of a human carcinoma in mice. *Proc Natl Acad Sci USA* **88**: 8616–8620

Brinkmann U, Buchner J and Pastan I (1992) Independent domain folding of *Pseudomonas* exotoxin and single-chain immunotoxins: influence of interdomain connections. *Proc Natl Acad Sci USA* **89**: 3075–3079

Brinkmann U, Reiter Y, Jung S, Lee B and Pastan I (1993) A recombinant immunotoxin containing a disulphide-stabilized Fv fragment. *Proc Natl Acad Sci USA* **90**: 7538–7542

Buchner J, Pastan I and Brinkmann U (1992) A method for increasing the yield of properly folded recombinant fusion proteins: single-chain immunotoxins from renaturation of bacterial inclusion bodies. *Anal Biochem* **205**: 263–270

Cafaro V, De Lorenzo C, Piccoli R, Bracale A, Mastronicola MR, Di Donato A and D'Alessio G (1995) The antitumor action of seminal ribonuclease and its quaternary conformations. *FEBS Lett* **359**: 31–34

Capasso S, Giordano F, Mattia CA, Mazzarella L and Zagari A (1983) A refinement of the structure of bovine seminal ribonuclease. *Biopolymers* **22**: 3227–3232

Chaudhary VK, Queen CP, Waldmann TA et al. (1989) A recombinant immunotoxin consisting of two antibody variable domains fused to *Pseudomonas* exotoxin. *Nature* **339**: 394–397

D'Alessio G, Di Donato A, Parente A and Piccoli R (1992) Seminal RNase-A unique member of the ribonuclease superfamily. *Trends Biochem Sci* **16**: 104–106

Deonarain MP and Epenetos AA (1994) Targeting enzymes for cancer therapy: old enzymes in new roles. *Br J Cancer* **70**: 786–794

Deonarain MP and Epenetos AA (1995) Construction, refolding and cytotoxicity of a scFv-seminal RNase fusion protein. *Tumour Targeting* **1**: 177–182

Deonarain MP, Rowlinson-Busza G, George AJT and Epenetos AA (1997) Anti-placental alkaline phosphatase single-chain Fv: Redesigned solubility, characterisation and in vivo tumour targeting. *Prot. Engineer* **10**: 89–98

Eagle H (1955) Propagation in fluid medium of a human eperdermoid carcinoma, strain KB. *Proc Soc Exp Biol Med* **89**: 362–364

Epenetos AA, Travers P, Gatter KC, Oliver RDT, Mason DY and Bodmer WF (1984) An immunohistological study of testicular germ cell tumours using two different monoclonal antibodies against placental alkaline phosphatase. *Br J Cancer* **49**: 11–15

Epenetos AA, Carr D, Johnson PM, Bodmer WF and Lavender JP (1986) Antibody-guided radiolocalisation of tumours in patients with testicular or ovarian cancer using two radioiodinated monoclonal antibodies to placental alkaline phosphatase. *Br J Radiol* **59**: 117–125

Ghetie V and Vitetta E (1994) Immunotoxins in the therapy of cancer: from bench to clinic. *Pharmacol Ther* **63**: 209–234

Glukhov BN, Jesusalimsky AP, Canter VM and Salganik RI (1976) Ribonuclease treatment of tick-borne encephalitis. *Arch Neurol* **33**: 598–602

Huston JS, McCartney J and Tai MS (1993) Medical applications of single-chain antibodies. *Int Rev Immunol* **10**: 195–217

Huston JS, George AJT, Tai M, McCartney JE, Jin D, Segal DM, Keck P and Opperman H (1994) In *Antibody Engineering: a Practical Approach*, Borrebaeck C. (ed). Oxford University Press: Oxford

Iles RK, Ind TE and Chard T (1994) Production of placental alkaline phosphatase (PLAP) and PLAP-like material by epithelial germ cell and non-germ cell tumours in vitro. *Br J Cancer* **69**: 274–278

Jost CR, Kurucz I, Jacobus CM, Titus JA, George AJ and Segal DM (1994) Mammalian expression and secretion of functional single-chain Fv molecules. *J Biol Chem* **269**: 26267–26273.

Kihara A and Pastan I (1994) Small chimeric toxins containing only transforming growth factor alpha and domain III of *Pseudomonas* exotoxin with good antitumor activity in mice. *Cancer Res* **54**: 5154–5159

Kim JS, Soucek J, Matousek J and Raines RT (1995a) Catalytic activity of bovine seminal ribonuclease is essential for its immunosuppressive and other biological activities. *Biochem J* **308**: 547–550

Kim JS, Soucek J, Matousek J and Raines RT (1995b) Mechanism of ribonuclease cytotoxicity. *J Biol Chem* **270**: 10525–10530

© Cancer Research Campaign 1998

Kunitz M (1946) A spectrophotometric method for the measurement of ribonuclease activity. *J Biol Chem* **164**: 563–568

Laccetti P, Portella G, Mastronicola MR, Russo A, Piccoli R, D'Alessio G and Vecchio G (1992) In vivo and in vitro growth-inhibitory effect of bovine seminal ribonuclease on a system of rat thyroid epithelial transformed cells and tumors. *Cancer Res* **52**: 4582–4589

Laccetti P, Spaletti-Cernia D, Portella G, De Corato P, D'Alessio G and Vecchio G (1994) Seminal ribonuclease inhibits tumour growth and reduces metastatic potential of Lewis lung carcinoma. *Cancer Res* **54**: 4253–4256

Mancheno JM, Gasset M, Onaderra M, Gavilanes JG and D'Alessio G (1994) Bovine seminal ribonuclease destabilises negatively-charged membranes. *Biochem Biophys Res Commun* **199**: 119–124

Matousek J, Soucek J, Riha J, Zankel TR and Benner SA (1995) Immunosuppressive activity of angiogenin in comparison with bovine seminal ribonuclease and pancreatic ribonuclease. *Comp Biochem Physiol B Biochem Mol Biol* **112**: 235–241

Murthy BS and Sirdeshmukh R (1992) Sensitivity of monomeric and dimeric forms of bovine seminal ribonuclease to human placental ribonuclease inhibitor. *Biochem J* **281**: 343–348

Newton D, Ilercil O, Laske DW, Oldfield E, Rybak SM and Youle RJ (1992) Cytotoxic ribonuclease chimeras: targeted tumoricidal activity in vitro and in vivo. *J Biol Chem* **267**: 19572–19578

Newton DL, Nicholls PJ, Rybak SM and Youle RJ (1994) Expression and characterisation of recombinant human eosinophil-derived neurotoxin and eosinophil-derived neurotoxin-anti-transferrin receptor sFv. *J Biol Chem* **269**: 26739–26745

O'Hare M, Brown AN, Hussain K, Gebhardt A, Watson G, Roberts LM, Vitetta ES, Thorpe PE and Lord JM (1990) Cytotoxicity of a recombinant ricin-A-chain fusion protein containing a proteolytically-cleavable spacer sequence. *FEBS Lett* **273**: 200–204

Parente A and D'Alessio G (1985) Reacquisition of quaternary structure by fully reduced and denatured seminal ribonuclease. *Eur J Biochem* **149**: 381–387

Pastan I, Chaudhary V and Fitzgerald DJ (1992) Recombinant toxins as a novel therapeutic agents. *Annu Rev Biochem* **61**: 331–354

Preuss K, Wagner S, Freudstein J and Scheit KH (1990) Cloning of cDNA encoding the complete precursor for bovine seminal ribonuclease. *Nucletc Acids Res* **18**: 1057–1058

Rybak SM, Saxena SK, Ackerman EJ and Youle RJ (1991) Cytotoxic potential of RNase and RNase hybrid proteins. *J Biol Chem* **266**: 21202–21207

Rybak SM, Hoogenboom HR, Meade HM, Raus JC, Schwartz D and Youle RJ (1992) Humanisation of immunotoxins. *Proc Natl Acad Sci USA* **89**: 3165–3169

Rybak SM, Pearson JW, Fogler WE, Volker K, Spence SE, Newton DL, Mikulski SM, Ardelt W, Riggs CW, Kung HF and Longo DL (1996) Enhancement of vincristine cytotoxicity in drug resistant cells by simultaneous treatment with onconase, an antitumor ribonuclease. *J Natl Cancer Inst* **88**: 747–753

Russo N, Denegris M, Didonato A and D'Alessio G (1993) Expression of native dimers of bovine seminal ribonuclease in an eukaryotic cell system. *FEBS Lett* **318**: 242–242

Sambrook J, Fritsch EF and Maniatis T (1989) In *Molecular Cloning: A Laboratory Manual*, 2nd edn. Cold Spring Harbor Laboratory Press: Cold Spring Harbor, NY

Savage P, Rowlinson-Busza G, Verhoeyen M, Spooner RA, So A, Windust J, Davies PJ and Epenetos AA (1993) Construction, characteristics and kinetics of a single-chain antibody recognising the tumour-associated antigen placental alkaline phosphatase. *Br J Cancer* **68**: 738–742

Spooner RA, Murray S, Rowlinson-Busza G, Deonarain MP, Chu A and Epenetos AA (1994) Genetically engineered antibodies for diagnostic pathology. *Hum Pathol* **25**: 606–614

Studier FW and Moffat BA (1986) Use of bacteriophage T7 RNA polymerase to direct selective high level expression of cloned genes. *J Mol Biol* **189**: 113–130

Toolan HW (1954) Transplantable human neoplasms maintained in cortisone treated laboratory animals. *Cancer Res* **14**: 660–666

Travers P and Bodmer W (1984) Preparation and characterisation of monoclonal antibodies against placental alkaline phosphatase and other human trophoblast-associated determinants. *Int J Cancer* **33**: 633–641

Vescia S, Tramontano D, Augusti-Tocco G and D'Alessio G (1980) In vitro studies on selective inhibition of tumor cell growth by seminal ribonuclease. *Cancer Res* **40**: 3740–3744

Wales R, Roberts LM and Lord JM (1993) Addition of an endoplasmic reticulum retrieval sequence to ricin A chain significantly increases cytotoxicity to mammalian cells. *J Biol Chem* **268**: 23986–23990

Winter G, Griffiths AD, Hawkins RE and Hoogenboom HR (1994) Making antibodies by phage display. *Annu Rev Imunol* **12**: 433–455

Youle RJ, Wu YN, Mikulski SM, Shogen K, Hamilton RS, Newton D, D'Alessio G and Gravell M (1994) RNase inhibition of human immunodeficiency virus infection of H9 cells. *Proc Natl Acad Sci USA* **91**: 6012–6016

*© Cancer Research Campaign 1998*